# Correlation Between Fixed-Luminance Flicker Full-Field Electroretinogram Response and Macular Cone Density in Healthy Individuals †

**DOI:** 10.3390/life15050694

**Published:** 2025-04-24

**Authors:** S. Saeed Mohammadi, Woong-Sun Yoo, Negin Yavari, Hassan Khojasteh Jafari, Christopher Or, Azadeh Mobasserian, Vahid Bazojoo, Amir Akhavanrezayat, Dalia El Feky, Osama Elaraby, Jia-Horung Hung, Cigdem Yasar, Ankur Gupta, Tanya Jain, Battuya Ganbold, Trung Ba Nguyen, Anadi Khatri, Zheng Xian Thng, Diana Do, Quan Dong Nguyen

**Affiliations:** 1 Spencer Center for Vision Research, Byers Eye Institute, Department of Ophthalmology, Stanford University, Palo Alto, CA 94303, USA; 2 Department of Ophthalmology, College of Medicine, Gyeongsang National University, Gyeongsang National University Hospital, Jinju 52727, Republic of Korea; 3 Bascom Palmer Eye Institute, University of Miami Miller School of Medicine, Miami, FL 33136, USA; 4 Department of Ophthalmology, Faculty of Medicine, Tanta University, Tanta 31527, Egypt; 5 Dr. Shroff Charity Eye Hospital, New Delhi 110002, India; 6 Bolor Melmii Eye Hospital, Ulaanbaatar 16050, Mongolia; 7 The Department of Ophthalmology, Viet Nam National Children’s Hospital, Hanoi 11512, Vietnam; 8 Department of Ophthalmology, Birat Medical College and Teaching Hospital, Kathmandu University, Biratnagar 45200, Nepal; 9 National Healthgroup Eye Institute, Tan Tock Seng Hospital, Singapore 308433, Singapore

**Keywords:** fixed-luminance flicker full-field electroretinogram, macular cone density, adaptive optics

## Abstract

This is the studyto investigate the correlation between macular cone density (MCD) and flicker electroretinogram (ERG) response in healthy eyes. In this exploratory study, 23 eyes from 12 healthy subjects were enrolled in this study. The fixed-luminance flicker full-field electroretinogram (ffERG) responses of the retina and MCDs at 24 locations were measured using the Diopsys^®^ NOVA™ system and the rtx1 adaptive optics retinal camera, respectively. Regression analysis was employed to evaluate the correlations. The mean age of the subjects was 30 ± 3 years. The average magnitudes of the flicker response and phase response were 13.44 ± 4.88 μV and 332.63 ± 22.12°, respectively. The MCDs for all 24 locations were 15,043 ± 3511 cones/mm². Among all locations, regression analysis revealed a significant correlation only at one specific location (0, −4°) between cone density and both the mean magnitude and phase of the flicker response, with *p*-values of 0.005 and 0.004, respectively.In conclusion, we identified a significant correlation between MCD and ffERG responses at a specific retinal locus (0, −4°). This finding may be attributed to the distribution of different cone types throughout the retina and the possibility that various cone types may contribute differently to ERG. Further studies are required to investigate this finding.

## 1. Introduction

The assessment of retinal function and its relationship to the underlying retinal structure plays a crucial role in diagnosing and monitoring various retinal disorders. One widely used tool to evaluate retinal function is the full-field electroretinogram (ffERG), which is a measure of the electrophysiological response of the entire retina to flashes of light [1]. By analyzing light-adapted 30 Hz flicker responses, the flicker ERG enables us to evaluate cone function in both hereditary and acquired retinal diseases, such as cone-rod dystrophy, achromatopsia, and diabetic retinopathy [2,3,4,5,6,7,8,9,10,11,12]. However, the conventional ERG recording method takes a long time and may not be suitable for all patients due to factors such as severe photophobia [13]. Diopsys^®^ NOVA™ (Diopsys^®^, Inc., Pine Brook, NJ, USA) is a patient-friendly, office-based device allowing for the assessment of retinal function. Unlike conventional ERG systems, which often require a dark room, Diopsys offers testing procedures that can be integrated into regular ophthalmic examinations, enhancing patient comfort and compliance [8].

The macula, specifically the fovea, plays a critical role in central high-acuity vision. The fovea is characterized by the absence of retinal vasculature, the excavation of the inner retina, and a lack of rod photoreceptors. It is also home to the highest density of cone photoreceptors in the human retina [14,15], which contributes to visual acuity and color vision [14,15,16]. Furthermore, the unique distribution of foveal cones, with a much higher density compared to peripheral cones, leads to an increased cortical sampling of foveal cones and a larger representation in the primary visual cortex [17,18]. The density of cones gradually decreases with increasing distance from the fovea, with the most substantial decline occurring within 1 to 2 mm of the fovea [19].

Adaptive optics (AO) systems minimize optical aberrations and enable the high-resolution imaging of retinal structures at a microscopic level. This technology allows for the study of cone density and arrangement at the macula, which is crucial for understanding both normal and pathological conditions of the retina [14,15].

Despite the essential roles of macular cone density (MCD) and the ERG in assessing retinal structure and function, the precise relationship between these two measures remains unclear. Establishing a correlation between MCD and flicker ERG responses may offer an alternative imaging biomarker not only for diagnosing and monitoring retinal diseases but also for evaluating, monitoring, and detecting age-related physiological changes and early pathological alterations associated with premature retinal degeneration.

This study aimed to investigate the relationship between MCD and fixed-luminance flicker ffERG responses using an AO fundus camera.

## 2. Methods

### 2.1. Study Design

This exploratory study was conducted from January 2023 to February 2023. All subjects underwent a comprehensive eye examination to exclude any ocular pathology. The inclusion criteria required participants to have a best-corrected visual acuity of 20/20 or better. Additionally, the spherical equivalent of their refraction had to fall within the range of +5.25 to −6.00 diopters to maintain consistency among participants. As this was an exploratory study, the sample size was not determined based on a formal statistical power calculation. Prior to the commencement of the research, institutional review board (IRB) approval was obtained from the Stanford IRB, ensuring compliance with ethical standards (IRB-41266). All participants were provided with detailed information about the study procedures, risks, and benefits, and a written consent form was obtained from all participants. The study adhered to the principles outlined in the Declaration of Helsinki.

### 2.2. ERG Recordings by Diopsys^®^ NOVA™ Fixed-Luminance Flicker Testing

The ffERG recordings were conducted using Diopsys^®^ NOVA™ (Diopsys, lnc., Middletown, PA, USA), an in-office ERG device, in a well-illuminated room free from any visual or auditory distractions. Standard procedures similar to those that have been detailed previously were followed to ensure consistent and accurate measurements [20]. To begin, the lower eyelids and forehead of the patient were thoroughly cleansed using alcohol swabs, with additional cleansing of the forehead using skin prep gel (NuPrep^®^, Weaver and Company, Aurora, CO, USA). Hypoallergenic skin electrodes were positioned below each lower eyelid, while a third electrode was attached to the forehead. The electrodes were then connected to the ERG device, with the contralateral eyelid electrode serving as a reference during recordings from either eye. The participant was instructed to hold the hand-held Mini Ganzfeld Stimulator over the eye being tested and maintain fixation on a center target with the other eye open. The full-field stimulus employed in the ffERG recording consisted of white flashes flickering at a frequency of 32 Hz over a white background. The luminosity of the white flash was set at 3 cd.s/m^2^ (600 cd/m^2^ as 5 ms flashes) over a white background of 30 cd/m^2^ adhering to the ISCEV standard protocol for ffERG [1]. Contamination by eye blinks or gross eye saccades was automatically rejected by the device through the exclusion of voltages exceeding a threshold of 50 μV [20,21].

Implicit time, which is reported in conventional ERG, refers to the duration from the onset of the stimulus to the peak of the relevant wave component [22]. Phase, as measured by Diopsys and reported in degrees, is related to implicit time, with a phase closer to 360 degrees corresponding to an implicit time closer to 0. Magnitude reflects the level of responsiveness of a subject’s retina to a light stimulus [23].

### 2.3. Macular Cone Density Recordings by the rtx1^TM^ Adaptive Optics Fundus Camera

**Figure 1:**
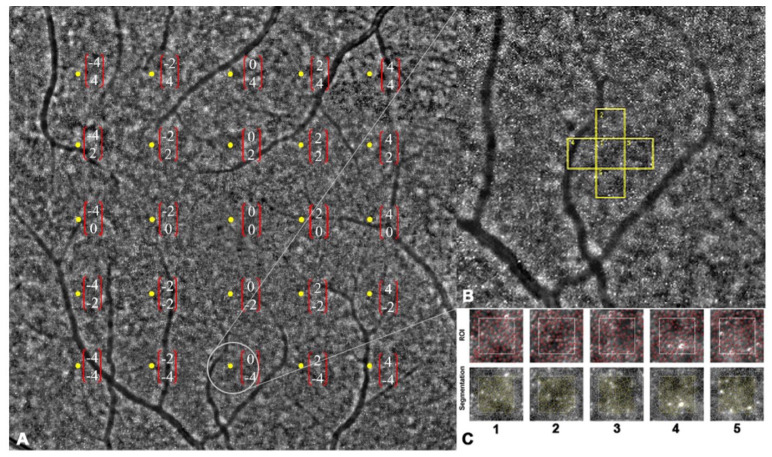
Montage of 24 captured images. (**A**) Macular cone density was measured in 24 predetermined locations (center locus was unmeasurable). (**B**,**C**) Regions of interest were segmented and analyzed by integrated software (followed by manual adjustment by an expert grader).

The assessment of macular cone density involved the utilization of high-resolution retinal imaging using the rtx1™ adaptive optics retinal flood-illumination camera (Imagine Eyes, Orsay, France). This noncontact “*en face*” imaging system is comprised of three main components: a high-resolution fundus camera, a Shack–Hartmann wave-front sensor, and a deformable mirror, enabling the real-time correction of ocular wavefront aberrations. Infrared illumination with a wavelength of 850 nm was employed, providing a resolution of approximately 2 μm. The field of view (FOV) captured was 4 × 4°, corresponding to approximately 1.2 × 1.2 mm on the retinal surface based on the axial length of the eye. Standardized imaging protocols were followed for acquiring images from the study eyes. Participants were instructed to fixate on an internal red fixation cross, which was monitored through a video camera incorporated into the rtx1^TM^ device to ensure proper fixation during image acquisition. A series of 40 images were captured over a duration of 4 s. Before image acquisition, the degree of adaptive optics correction displayed on the camera software panel was confirmed to be less than 1 milliradian, ensuring the effective correction of optical aberrations and improved image quality [17]. The cone mosaic was imaged at 24 center points surrounding the fovea, where the maximum cone density is found, spaced 2 degrees apart vertically and horizontally (Figure 1A). The regions of interest (ROIs), corresponding to central vision and including 2° and 4° eccentricities along the horizontal and vertical meridians (the center locus was unmeasurable), were montaged afterward to evaluate the accuracy of the captured images. The foveal reference point for each patient was determined by identifying the central point of the image acquired when the patient fixated on an internal fixation cross set at 0° angle (x = 0° and y = 0°). Eccentricity along the orthogonal meridians was measured as the distance between the ROIs and the foveal reference point [24].

Retinal images were acquired and assessed for image quality using AO Image (Imagine Eyes, Orsay, France). Then, the acquired images were processed using proprietary software AO detect (Imagine Eyes, Orsay, France) provided by the manufacturer. This processing involved generating a high-contrast image of the retina within a 4 × 4° area, enhancing the signal-to-noise ratio, and achieving a resolution of 0.8 μm/pixel (Figure 1B,C). To avoid blood vessels, the ROIs could be shifted within a range of 50 μm, if necessary [25]. Cone cells in each location were automatically detected using the AOdetect software (ver. 3.0), followed by manual adjustment by an expert grader (A.A.). Cone density in each specific location was calculated by measuring the mean density in five adjacent central ROIs.

### 2.4. Statistical Analysis

The collected data were analyzed using IBM SPSS Statistics for Windows, version 23 (IBM Corp., Armonk, NY, USA). The mean and standard deviations were employed to describe the statistical measures, and the unpaired sample t-test was utilized to identify any significant differences between groups. Pearson’s correlation was used to evaluate the relationship. In all statistical analyses, a probability value of 0.05 was considered the threshold for significance.

## 3. Results

Our study included 23 eyes of 12 healthy subjects. Demographic information is included in Table 1.

**Figure 2:**
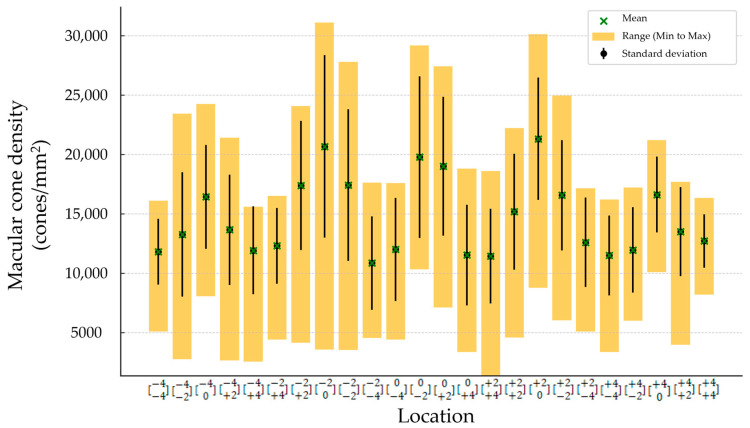
Macular cone density in each location.

**Figure 3:**
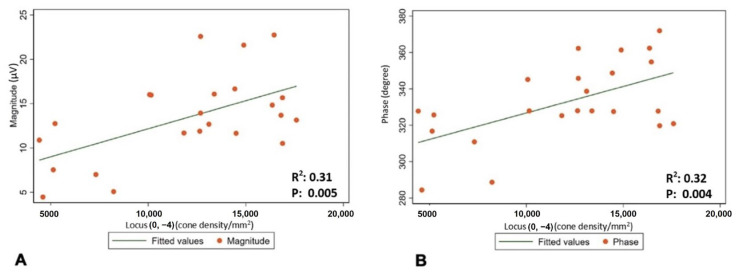
(**A**) Correlation between cone density in location $\left[\begin{array}{c} 0\\ -4 \end{array}\right]$ and mean magnitude of flicker response. (**B**) Correlation between cone density in location $\left[\begin{array}{c} 0\\ -4 \end{array}\right]$ and mean phase of flicker response.

The mean (±SD) spherical equivalent (SE) was −0.48 (±0.23) D. The mean (±SD) magnitude of flicker response was 13.44 ± 4.88 µV, and the mean (±SD) phase of the flicker response was 332.63 ± 22.12 °. The total mean (±SD) was 14,648.64 cones/mm^2^ (±5489.59). Minimum number of cones was found to be in location $\left[\begin{array}{c} -2\\ -4 \end{array}\right]$, and it was 10,858 (±3945.52). The maximum number of cones was found to be in location $\left[\begin{array}{c} +2\\ 0 \end{array}\right]$, and it was 21,324.32 (±5162.36) (Figure 2 and Table 2). Regression analysis was performed to evaluate correlation between the magnitude and phase of the ffERG and macular cone density and showed a significant correlation in location $\left[\begin{array}{c} 0\\ -4 \end{array}\right]$ (*p*-value 0.005 and 0.004, and R^2^ 0.31 and 0.32, respectively) (Table 3 and Figure 3A,B).

## 4. Discussion

The observed correlation between the macular cone density and magnitude and phase of Diopsys fixed-luminance flicker full-field ERG (ffERG) carries promising clinical implications. Full-field flicker ERG is a comprehensive method to evaluate cone cell function throughout the retina, but its limitations in terms of time consumption and patient discomfort, especially for those with photophobia, necessitate the evaluation of alternative approaches. Our results showed a statistically significant correlation between macular cone density at location $\left[\begin{array}{c} 0\\ -4 \end{array}\right]$ and fixed-luminance flicker ffERG parameters. This could be associated with the unique organization of the human trichromatic cone mosaic, the fact that the signal from the M cone contributes more to the ERG than the signal from other cones, and the distribution of various types of cones throughout the retina [26].

By establishing a statistically significant correlation between macular cone density at location $\left[\begin{array}{c} 0\\ -4 \end{array}\right]$ and fixed-luminance flicker ffERG parameters, our findings suggest a potential link between the structural characteristics of the macular cones and their functional performance, as assessed by the flicker ffERG, and provide a promising avenue for early diagnosis and the monitoring of retinal diseases that affect cone function.

By establishing a relationship between the macular cone density and magnitude and phase of the fixed-luminance flicker ffERG, clinicians may be able to identify subtle and localized changes in cone density even before the onset of noticeable symptoms or significant functional impairments. These changes might not have a substantial impact on the flicker ffERG; however, they can be identified through rtx1^TM^. This early detection could be crucial in initiating timely interventions and treatment strategies, ultimately leading to better patient outcomes. Moreover, the correlation between the macular cone density and magnitude and phase of flicker ffERG provides a non-invasive and relatively quick alternative for assessing cone function compared to flicker ffERG techniques. Flicker ffERG can be time-consuming, requires high level of patient compliance, and involves exposing patients to bright flashes of light, which can be challenging for individuals with photophobia or those who may have difficulty tolerating such stimuli. By utilizing the rtx1^TM^ measurement, which are less invasive and more patient-friendly, clinicians can potentially overcome these limitations and expand the accessibility of diagnostic assessments for a broader range of patients.

This finding also opens possibilities for further research and advancements in understanding retinal diseases. By exploring the relationship between macular cone density and flicker ffERG in different patient populations and disease conditions, researchers can gain insights into the underlying mechanisms of cone dysfunction.

While the findings of the study provide valuable insights into the relationship between these variables, it is important to acknowledge the limitations that may have influenced the results and interpretation of the findings.

Firstly, the small sample size used in the study limits the generalizability of the results. With a limited number of participants, the findings may not adequately represent the broader population or allow for robust statistical analyses. As this was an exploratory study, a formal sample size calculation to ensure statistical power was not performed. Secondly, the mean age of the studied subjects was 35.2 years, as participants were selected based on their health status to exclude systemic diseases. This preference for younger, healthy individuals introduces a potential selection bias and may limit the applicability of the findings to older populations or those with systemic conditions. Thirdly, the study was conducted in a single tertiary hospital located in the western United States, limiting the ethnic diversity of the study population. As the findings may not fully represent populations from other geographic regions or ethnic backgrounds. Future research with larger sample sizes, including diseased retinas and incorporating multicenter and multinational studies, would be necessary to confirm the observed correlations, provide a more comprehensive evaluation of these relationships, and enhance the reliability of the findings.

Another limitation of the study lies in the subjective nature of the rtx1^TM^ measurement technique for determining the foveal center point. The identification of the foveal center point relies on the operator’s skills and the patient’s compliance, introducing potential variability and measurement errors. Different operators may have different levels of expertise or interpretations, leading to inconsistencies in identifying the exact foveal center point. Consequently, such a subjective aspect could introduce bias and impact the accuracy of the cone density measurements.

Furthermore, the assumption made by the rtx1^TM^ device, which considers the center of the image as the center of the fovea, may not always align with the true anatomical center of the fovea [27]. This discrepancy introduces a potential source of error in determining the exact location for cone density measurements. Variations in the actual position of the foveal center among individuals could affect the correlation results between the cone density and the Diopsys flicker ffERG variables. If the patient maintains fixation on the target and sustains it throughout the study, the discrepancy may not have a significant impact on the results. However, even subtle instances of fixation loss during the imaging process could potentially have a significant effect on the results.

Additionally, the study identified the subjective and operator-dependent nature of adjusting the focus for each image as a limitation [28]. Achieving optimal focus for every image is crucial to obtain accurate measurements of cone density. However, differences in operator technique or interpretation may result in variations in focus adjustments, potentially leading to inconsistencies and affecting the precision of the cone density measurements. The lack of standardized protocols for adjusting focus across different operators further exacerbates this limitation.

Considering these limitations, it is important to interpret the findings of this study with caution. While the observed correlations between the macular cone density at location $\left[\begin{array}{c} 0\\ -4 \end{array}\right]$ and magnitude and phase of Diopsys flicker ffERG provide initial evidence of a relationship, the small sample size, subjective nature of the rtx1^TM^ measurements, reliance on operator skills, and potential inconsistencies in determining the foveal center point and adjusting focus introduce uncertainties.

Future research should aim to address these limitations by employing larger sample sizes, implementing standardized protocols for measurements, and exploring alternative objective techniques for determining the foveal center. By addressing these limitations, future studies can enhance the reliability and validity of the findings, providing a more comprehensive understanding of the relationship between macular cone density and Diopsys flicker ffERG variables.

## 5. Conclusions

The observed correlation between the macular cone density in location $\left[\begin{array}{c} 0\\ -4 \end{array}\right]$ and magnitude and phase of Diopsys full-field flicker ERG may hold important clinical implications for the diagnosis and monitoring of retinal diseases affecting the cone. The correlation also offers a non-invasive and relatively quick alternative to traditional ERG techniques, which can be time-consuming and difficult for certain patients. Future research is necessary to enhance our understanding of the relationship between macular cone density and full-field flicker ERG variables.

## Figures and Tables

**Table 1:** Demographic characteristics of study subjects.

Age (±SD)		30.5 Years (3.2)
Gender	Female	5 (41.6%)
	Male	7 (58.3%)
Race/Ethnicity	White/Caucasian	6 (50.0%)
	Asian	4 (33.3%)
	Latin/Hispanic	1 (8.33%)
	African American	1 (8.33%)

**Table 2:** Mean (±SD), minimum, and maximum measurements of macular cone density at 24 locations.

Location	Mean	±SD	Minimum	Maximum
$\left[\begin{array}{c} -4\\ -4 \end{array}\right]$	11,813.20	2779.05	5085.70	16,115.60
$\left[\begin{array}{c} -4\\ -2 \end{array}\right]$	13,275.42	5247.00	2757.50	23,441.60
$\left[\begin{array}{c} -4\\ 0 \end{array}\right]$	16,436.55	4372.85	8060.80	24,255.80
$\left[\begin{array}{c} -4\\ +2 \end{array}\right]$	13,661.06	4640.54	2651.50	21,405.20
$\left[\begin{array}{c} -4\\ +4 \end{array}\right]$	11,922.88	3701.89	2540.00	15,609.00
$\left[\begin{array}{c} -2\\ +4 \end{array}\right]$	12,312.16	3187.89	4418.00	16,513.20
$\left[\begin{array}{c} -2\\ +2 \end{array}\right]$	17,401.92	5447.58	4161.40	24,087.40
$\left[\begin{array}{c} -2\\ 0 \end{array}\right]$	20,682.61	7684.33	3561.80	31,117.00
$\left[\begin{array}{c} -2\\ -2 \end{array}\right]$	17,428.26	6395.86	3520.50	27,810.60
$\left[\begin{array}{c} -2\\ -4 \end{array}\right]$	10,858.00	3945.52	4535.20	17,639.60
$\left[\begin{array}{c} 0\\ -4 \end{array}\right]$	12,009.63	4339.77	4417.80	17,590.00
$\left[\begin{array}{c} 0\\ -2 \end{array}\right]$	19,781.84	6808.06	10,321.60	29,193.60
$\left[\begin{array}{c} 0\\ +2 \end{array}\right]$	19,012.60	5838.91	7112.40	27,434.40
$\left[\begin{array}{c} 0\\ +4 \end{array}\right]$	11,533.60	4237.96	3378.20	18,804.40
$\left[\begin{array}{c} +2\\ +4 \end{array}\right]$	11,433.50	3990.50	1373.40	18,617.80
$\left[\begin{array}{c} +2\\ +2 \end{array}\right]$	15,186.53	4889.59	4579.60	22,233.80
$\left[\begin{array}{c} +2\\ 0 \end{array}\right]$	21,324.32	5162.36	8789.40	30,124.60
$\left[\begin{array}{c} +2\\ -2 \end{array}\right]$	16,574.20	4652.68	6042.60	24,979.20
$\left[\begin{array}{c} +2\\ -4 \end{array}\right]$	12,608.91	3775.08	5076.80	17,149.40
$\left[\begin{array}{c} +4\\ -4 \end{array}\right]$	11,496.59	3355.76	3374.60	16,214.20
$\left[\begin{array}{c} +4\\ -2 \end{array}\right]$	11,955.00	3598.36	5993.00	17,240.60
$\left[\begin{array}{c} +4\\ 0 \end{array}\right]$	16,625.06	3200.03	10,106.40	21,209.80
$\left[\begin{array}{c} +4\\ +2 \end{array}\right]$	13,508.07	3765.50	3964.80	17,711.80
$\left[\begin{array}{c} +4\\ +4 \end{array}\right]$	12,725.42	2248.25	8189.40	16,333.40

**Table 3:** Correlation between magnitude and phase of ffERG and cone density at 24 locations.

Location		R^2^	*p*-Value
$\left[\begin{array}{c} -4\\ -4 \end{array}\right]$	Magnitude	0.321	0.425
	Phase	0.010	0.658
$\left[\begin{array}{c} -4\\ -2 \end{array}\right]$	Magnitude	0.150	0.068
	Phase	0.127	0.094
$\left[\begin{array}{c} -4\\ 0 \end{array}\right]$	Magnitude	0.000	0.915
	Phase	0.000	0.912
$\left[\begin{array}{c} -4\\ +2 \end{array}\right]$	Magnitude	0.000	0.992
	Phase	0.001	0.882
$\left[\begin{array}{c} -4\\ +4 \end{array}\right]$	Magnitude	0.149	0.076
	Phase	0.111	0.129
$\left[\begin{array}{c} -2\\ +4 \end{array}\right]$	Magnitude	0.021	0.513
	Phase	0.113	0.125
$\left[\begin{array}{c} -2\\ +2 \end{array}\right]$	Magnitude	0.011	0.631
	Phase	0.029	0.442
$\left[\begin{array}{c} -2\\ 0 \end{array}\right]$	Magnitude	0.001	0.856
	Phase	0.005	0.763
$\left[\begin{array}{c} -2\\ -2 \end{array}\right]$	Magnitude	0.018	0.562
	Phase	0.032	0.432
$\left[\begin{array}{c} -2\\ -4 \end{array}\right]$	Magnitude	0.035	0.387
	Phase	0.104	0.132
$\left[\begin{array}{c} 0\\ -4 \end{array}\right]$	Magnitude	0.315	0.005
	Phase	0.326	0.004
$\left[\begin{array}{c} 0\\ -2 \end{array}\right]$	Magnitude	0.101	0.170
	Phase	0.135	0.110
$\left[\begin{array}{c} 0\\ +2 \end{array}\right]$	Magnitude	0.029	0.455
	Phase	0.001	0.854
$\left[\begin{array}{c} 0\\ +4 \end{array}\right]$	Magnitude	0.004	0.756
	Phase	0.002	0.834
$\left[\begin{array}{c} +2\\ +4 \end{array}\right]$	Magnitude	0.002	0.842
	Phase	0.000	0.932
$\left[\begin{array}{c} +2\\ +2 \end{array}\right]$	Magnitude	0.037	0.379
	Phase	0.004	0.769
$\left[\begin{array}{c} +2\\ 0 \end{array}\right]$	Magnitude	0.065	0.252
	Phase	0.055	0.291
$\left[\begin{array}{c} +2\\ -2 \end{array}\right]$	Magnitude	0.022	0.492
	Phase	0.046	0.322
$\left[\begin{array}{c} +2\\ -4 \end{array}\right]$	Magnitude	0.051	0.325
	Phase	0.133	0.103
$\left[\begin{array}{c} +4\\ -4 \end{array}\right]$	Magnitude	0.076	0.212
	Phase	0.110	0.131
$\left[\begin{array}{c} +4\\ -2 \end{array}\right]$	Magnitude	0.039	0.366
	Phase	0.036	0.381
$\left[\begin{array}{c} +4\\ 0 \end{array}\right]$	Magnitude	0.001	0.851
	Phase	0.011	0.621
$\left[\begin{array}{c} +4\\ +2 \end{array}\right]$	Magnitude	0.000	0.897
	Phase	0.000	0.918
$\left[\begin{array}{c} +4\\ +4 \end{array}\right]$	Magnitude	0.014	0.579
	Phase	0.032	0.409

## Data Availability

The data presented in this study are available in this article.
